# Distinct blood inflammatory biomarker clusters stratify host phenotypes during the middle phase of COVID-19

**DOI:** 10.1038/s41598-022-26965-7

**Published:** 2022-12-28

**Authors:** Paul W. Blair, Joost Brandsma, Josh Chenoweth, Stephanie A. Richard, Nusrat J. Epsi, Rittal Mehta, Deborah Striegel, Emily G. Clemens, Sultanah Alharthi, David A. Lindholm, Ryan C. Maves, Derek T. Larson, Katrin Mende, Rhonda E. Colombo, Anuradha Ganesan, Tahaniyat Lalani, Christopher J. Colombo, Allison A. Malloy, Andrew L. Snow, Kevin L. Schully, Charlotte Lanteri, Mark P. Simons, John S. Dumler, David Tribble, Timothy Burgess, Simon Pollett, Brian K. Agan, Danielle V. Clark, the EPICC COVID-19 Cohort Study Group, J. Cowden, M. Darling, T. Merritt, T. Wellington, A. Rutt, C. Conlon, P. Faestel, C. Mount, A. Smith, R. Tant, T. Warkentien, C. Berjohn, G. Utz, C. Madar, C. Uyehara, K. Chung, C. English, C. Fox, M. Grother, P. Hickey, E. Laing, J. Livezey, E. Parmelee, J. Rozman, M. Sanchez, A. Scher, T. Chao, R. Chapleau, A. Fries, K. Reynolds, D. Hostler, J. Hostler, K. Lago, C. Maldonado, T. Hunter, R. Mody, M. Wayman, N. Huprikar

**Affiliations:** 1 The Henry M. Jackson Foundation for the Advancement of Military Medicine, Inc., 6720A Rockledge Dr, Bethesda, MD 20817 USA; 2 Department of Pathology, Uniformed Services University of the Health Sciences, Bethesda, MD USA; 3 Infectious Disease Clinical Research Program, Department of Preventive Medicine and Biostatistics, Uniformed Services University of the Health Sciences, Bethesda, MD USA; 4 Department of Medicine, Uniformed Services University of the Health Sciences, Bethesda, MD USA; 5 Brooke Army Medical Center, Joint Base San Antonio-Ft Sam Houston, San Antonio, TX USA; 6 Departments of Internal Medicine and Anesthesiology, Wake Forest School of Medicine, Winston-Salem, NC USA; 7 Naval Medical Center San Diego, San Diego, CA USA; 8 Fort Belvoir Community Hospital, Fort Belvoir, VA USA; 9 Madigan Army Medical Center, Joint Base Lewis-McChord, Tacoma, WA USA; 10 Walter Reed National Military Medical Center, Bethesda, MD USA; 11 Naval Medical Center Portsmouth, Portsmouth, VA USA; 12 Department of Pediatrics, Uniformed Services University of the Health Sciences, Bethesda, MD USA; 13 Department of Pharmacology & Molecular Therapeutics, Uniformed Services University of the Health Sciences, Bethesda, MD USA; 14 Biological Defense Research Directorate, Naval Medical Research Center-Frederick, Ft. Detrick, MD USA; 15 Tripler Army Medical Center, Honolulu, HI USA; 16 Uniformed Services University of the Health Sciences, Bethesda, MD USA; 17 United States Air Force School of Aerospace Medicine, Dayton, OH USA; 18 Womack Army Medical Center, Fort Bragg, NC USA; 19 William Beaumont Army Medical Center, El Paso, TX USA

**Keywords:** Biomarkers, Computational biology and bioinformatics, Cytokines, Inflammation, Viral infection

## Abstract

The associations between clinical phenotypes of coronavirus disease 2019 (COVID-19) and the host inflammatory response during the transition from peak illness to convalescence are not yet well understood. Blood plasma samples were collected from 129 adult SARS-CoV-2 positive inpatient and outpatient participants between April 2020 and January 2021, in a multi-center prospective cohort study at 8 military hospitals across the United States. Plasma inflammatory protein biomarkers were measured in samples from 15 to 28 days post symptom onset. Topological Data Analysis (TDA) was used to identify patterns of inflammation, and associations with peak severity (outpatient, hospitalized, ICU admission or death), Charlson Comorbidity Index (CCI), and body mass index (BMI) were evaluated using logistic regression. The study population (n = 129, 33.3% female, median 41.3 years of age) included 77 outpatient, 31 inpatient, 16 ICU-level, and 5 fatal cases. Three distinct inflammatory biomarker clusters were identified and were associated with significant differences in peak disease severity (p < 0.001), age (p < 0.001), BMI (p < 0.001), and CCI (p = 0.001). Host-biomarker profiles stratified a heterogeneous population of COVID-19 patients during the transition from peak illness to convalescence, and these distinct inflammatory patterns were associated with comorbid disease and severe illness due to COVID-19.

## Introduction

While clinical risk factors for coronavirus disease 2019 (COVID-19) severity have been described, mechanisms of inflammation associated with these baseline clinical features are less well understood^1^. SARS-CoV-2 infections range from asymptomatic to fatal illness, and this spectrum is associated with host risk factors such as age and chronic noncommunicable disease (NCD), including obesity and cardiovascular disease^2^. However, the pathways from host factors to COVID-19 severity and sequelae are largely unknown. Given the heterogeneity of COVID-19 severity and a growing immunomodulatory treatment armamentarium^2,3^, pathologic inflammation patterns and their association with comorbidities need to be identified to optimize treatment selection.

COVID-19 severity and inflammation occur in three broadly-defined phases. The “acute phase” is associated with peak disease severity and maximum levels of inflammatory host-biomarkers, and generally occurs during the first 2 weeks of illness. The transition between the “acute” and “late” post-COVID phase is less well-defined, but generally occurs between 15- and 28-days post symptom onset (dpso)^4,5^. While this is characterized by persistent inflammation in severely ill individuals, this period of recovery and convalescence is referred to herein as the “middle”, rather than “inflammatory” phase, to include disease resolution in less severe COVID-19 disease courses. While inflammation may subside in mild cases, persistently high pro-inflammatory cytokines have been noted in severe cases during this period. This time window of wide differences in the immune response may be best suited to elucidate the relationship between host factors and severe COVID-19. In silico stratification of host-biomarker profiles has the potential to identify distinct phenotypes associated with disease severity and patient comorbidities, which can in turn lead to of the development of more personalized treatment approaches.

We hypothesized that clustering of blood inflammatory biomarker profiles would identify unique phenotypes during the middle phase of COVID-19, that are associated with differences in severity, demographics, and co-morbid conditions known to predispose patients to worse outcomes during SARS-CoV-2 infection^4^. Our analysis used samples collected during the 15–28 dpso period from an observational, multi-center cohort of participants with mild to severe COVID-19 at US military treatment facilities. Protein analytes that were measured included biomarkers of vascular damage, organ injury, and Th1-type immune mediators, that were selected from prior unpublished analyses of non-COVID-19 sepsis^6^, as well as biomarkers in general clinical use^7^. Our objective was to stratify the inflammatory response during the middle phase of COVID-19, and to explore associations between biomarker clusters and demographic characteristics, baseline comorbidities, and peak severity of COVID-19.

## Methods

Participants were enrolled in a prospective, multi-center COVID-19 cohort under the Epidemiology, Immunology, and Clinical Characteristics of Emerging Infectious Diseases with Pandemic Potential (EPICC) protocol, at 8 military treatment facilities (Brooke Army Medical Center, San Antonio, TX; Fort Belvoir Community Hospital, Fort Belvoir, VA; Madigan Army Medical Center, Joint Base Lewis-McChord, WA; Naval Medical Center Portsmouth, Portsmouth, VA; Naval Medical Center San Diego, San Diego, CA; Tripler Army Medical Center, Honolulu, HI; William Beaumont Army Medical Center, El Paso, TX; Walter Reed National Military Medical Center, Bethesda, MD) between April 2020 and January 2021^8^. The protocol was approved by the Uniformed Services University Institutional Review Board (IDCRP-085)^9^. All patients provided written informed consent and all procedures were performed in accordance with the ethical standards of the Helsinki Declaration of the World Medical Association. EPICC study enrollment included subjects ≥ 18 years of age with laboratory-confirmed or suspected SARS-CoV-2 infection seeking inpatient or outpatient medical care. Following consent, demographic, comorbidity, and illness data were collected through participant interviews and a review of the participant’s electronic medical record or using participant completed surveys implemented in November 2020. Subjects with a positive clinical SARS-CoV-2 RT-PCR result and plasma samples collected were included in this analysis. Results of well-described^10^ COVID-19 clinical biomarkers CRP, ferritin, and IL-6, were explored from 217 participants with plasma collected 0–29 days post symptom onset (dpso) to determine if the longitudinal inflammatory biomarker LOESS (locally estimated scatterplot smoothing) curve peaked between 14 to 28 days per previously published phases of illness framework for studying COVID-19 (Supplementary Fig. S1)^4^. Subsequent analyses were restricted to the 129 participants with samples collected during the middle phase defined as 15–28 dpso. Receipt of baricitinib, tocilizumab, hydroxychloroquine, or systemic steroids (equivalent to prednisone 10 mg daily or above) at the time of blood collection was determined through the electronic medical record or participant surveys.

Plasma samples were prospectively collected after enrollment as previously described^9^. Venous whole blood samples were centrifuged for 10 min at 1500*g* and collected plasma was stored at − 80 °C. A panel of 12 inflammatory proteins were measured in the plasma samples using the high dynamic range automated enzyme-linked immunosorbent assay Ella microfluidic analyzer (ProteinSimple, San Jose, California, USA) (see Supplemental Methods). Analytes included: IL-6, CXCL10, IL-1RA, d-dimer, procalcitonin, ferritin, VEGF-A, IL-5, soluble receptor for advanced glycation end-product (RAGE), TNFR1, IFN-γ, and C-reactive protein (CRP). These analytes were selected to include biomarkers in clinical use for prognostication (i.e., CRP, procalcitonin, ferritin, and d-dimer)^7^, based on prior COVID-19 literature (i.e., IL-6, IFN-γ and CXCL10)^11^, and identified to be representative of prior TDA-based non-COVID-19 sepsis clusters (i.e., IL-1RA, VEGF-A, IL-5, RAGE, and TNFR1)^6,12,13^. All protein concentrations were log_10_-transformed and normalized for site-to-site variation using the *R* package *SVA ComBat*^14^. A small number (1.6%) of missing values were imputed using a *k*-nearest neighbor model, and out-of-range values were imputed using either the lowest or highest measured value within range of the Ella platform. Correlation between analytes was explored with a principal component analysis and determining the Spearman’s correlation coefficients. For subjects (N = 22) with two or three samples available from different timepoints, the sample with the highest variability (coefficient of variation) was selected per subject to optimize cluster identification^15^. A sensitivity analysis was performed to determine the effect on cluster affiliation using the collection time with the highest rank across analytes rather than the highest coefficient of variation.

Herein we define inflammatory host-biomarker phenotypes of COVID-19 identified by Topological Data Analysis (TDA) and associated comorbid conditions and disease severity. TDA is a multivariate pattern analytical tool that uses an unsupervised approach to dimensionality reduction and data visualization^16^. A key advantage of TDA over other dimensionality reduction techniques, such as principal component analysis, is that it is not limited to 2 axes and is less sensitive to loss of information^17^. TDA can be used to identify phenotype-biomarker relationships^17–19^ and has previously identified patient subgroups that could benefit from personalized interventions for heterogenous noncommunicable diseases^16,18^. Protein expression networks were generated solely using biomarkers levels with the TDA “Mapper” algorithm using the EurekaAI platform (SymphonyAI, Los Altos, CA, USA)^17,20,21^. TDA networks were generated for a range of resolution settings to examine the persistence of subject clusters and their interrelatedness (see Supplemental Methods). Peak severity (outpatient, hospitalized, ICU-level or death) color gradients were overlaid on identified clusters. Levels of the individual proteins in each TDA group were summarized in a series of boxplots (*R* package “ggplot2” v3.3.5). Backward selection stepwise logistic regression using a Bernoulli-adjusted significance level of 0.0042 (i.e., 0.05/12) was used to identify which proteins were up- or downregulated within each cluster. While TDA clusters will inherently have different biomarker levels, this was performed to simplify inference about representative biomarkers and for future validation in external cohorts. A sensitivity analysis was performed adjusting for peak severity to determine the effect of covariate selection. An additional sensitivity analysis was performed excluding participants receiving systemic steroids.

Summary statistics were calculated for the clusters, comparing baseline demographics (e.g., sex, age, race, ethnicity, selected medical comorbidities), days post symptom onset, peak severity, steroid use, and the inflammatory biomarkers by clusters using either Chi-square (categorical values), Fisher exact (categorical values), or Mann–Whitney U tests (continuous values). Charlson Comorbidity Index (CCI) and body mass index (BMI) values were divided into score-based categories (i.e., CCI: 0, 1–2, 3–4, or 5+; BMI: < 30, 30–39.9, or ≥ 40 kg/m^2^) to describe the prevalence of comorbid conditions by cluster on a bar plot but were otherwise treated as continuous values. BMI values were not available from 6.2% of the cohort. Peak severity was categorized for each participant [outpatient, non-ICU (intensive care unit) inpatient, and ICU or death]. Multivariable logistic regression adjusting for peak severity was used to identify associations between each cluster and BMI or CCI at a significance level of 0.05. A sensitivity analysis was performed to adjust for duration of symptoms at sample collection. Additionally, logistic regression models to examine the association between clusters and death or ICU care at peak illness were performed. Area under the receiver operating characteristic curves (AUROC) and Akaike information criterion (AIC; measure of model parsimoniousness) estimates were compared between models with and without adjustment for baseline demographics (i.e., age, sex, and CCI), clinical biomarkers (i.e., d-dimer, ferritin, and CRP), and cluster covariates. All statistical analyses were performed in Stata (version 15.0; StataCorp LLC, College Station, TX, USA) and R version 4.0.2^22^.

## Results

**Figure 1:**
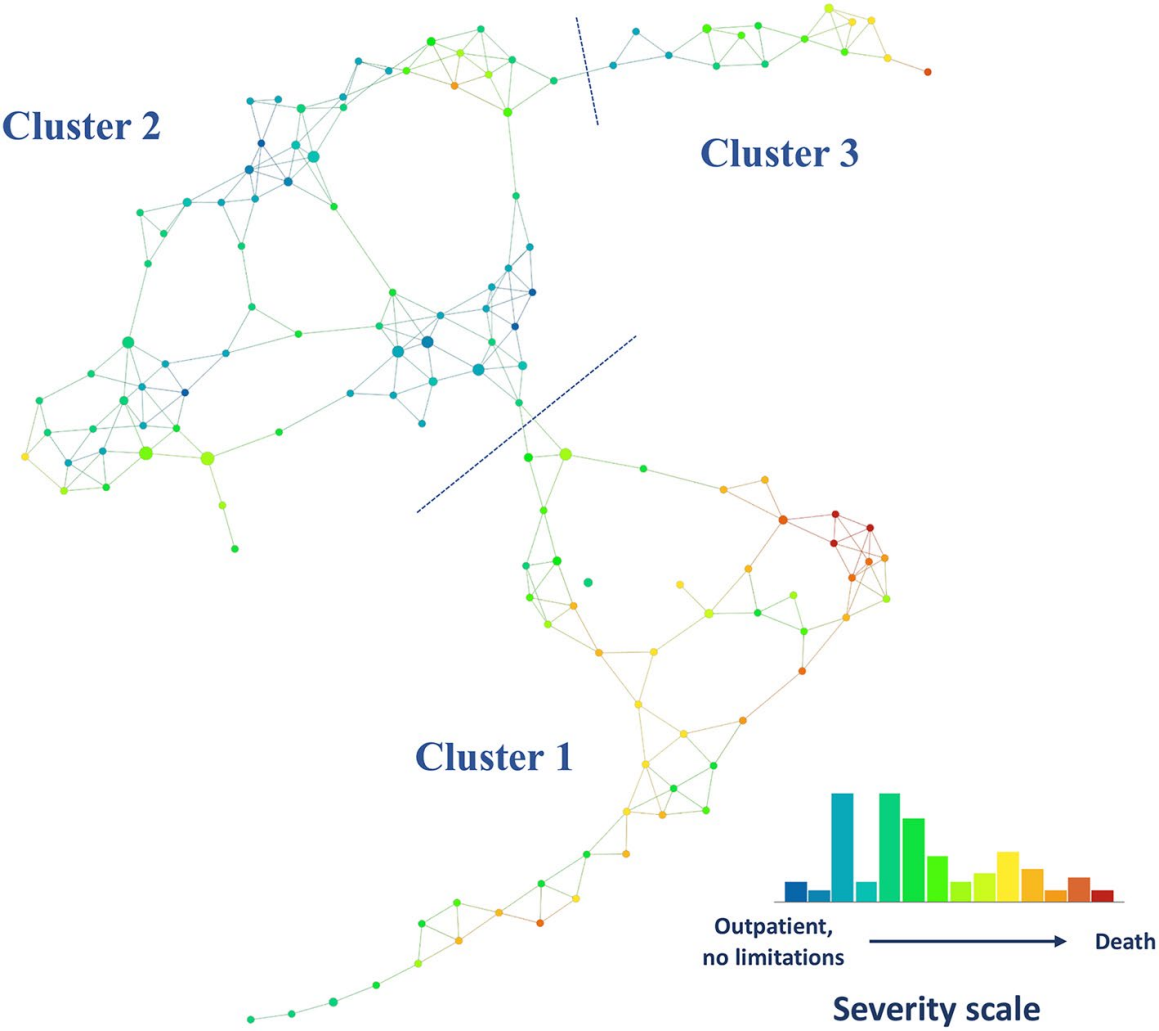
Topological data analysis (TDA) network of protein expression during the middle-phase of COVID-19. Distinct protein expression phenotypes (Clusters 1, 2, and 3) were identified based on density and break points in the network and persistence of the clusters. Each node represents a combination of 12 plasma protein analyte levels and its size increases with the number of participants that are included. Edges (lines between nodes) indicate that patients are represented in more than one node. The network is colored by the average score on the disease severity scale (from outpatients without limitations [green] to death [red]) in each node. Analysis was performed on the EurekaAI Workbench (SymphonyAI, Los Altos, CA, USA).

Biomarkers CRP, IL-6, and ferritin were stratified by severity and explored for the 249 participants in the EPICC cohort between 0 and 28 dpso using a scatter plot with LOESS (locally estimated scatterplot smoothing) curves. This demonstrated average cytokines peaked or remained elevated during the described middle phase (15–28 dpso) among ICU-level or fatal courses of illness (Supplementary Fig. S1). Based on these findings and clinical frameworks of illness^23^, we restricted our analysis to participants that had blood collected within the middle phase. As participants were enrolled at different durations post-symptom onset, our analysis included 129 participants (66.7% male, median 41.3 years of age) including 77 outpatient, 31 inpatient, 16 ICU-level, and 5 fatal cases (Table 1) between 15 to 28 days of illness. Correlation along a PCA axis was observed among procalcitonin, TNFR1, IL-6, CRP, and IL-1RA while RAGE, IFN-γ, IL-5, and VEGF-A were less correlated with the other analytes. Additionally, variance increased with each level of peak severity (Supplementary Fig. S2). These results supported the additive information provided by the 12 protein analytes, and TDA was performed. Interestingly, 3 distinct inflammatory proteins clusters, labeled Cluster 1, Cluster 2, and Cluster 3 (Fig. 1; Supplementary Fig. S3), were consistently identified using TDA. There was no obvious difference between Clusters 2 and 3 by PCA alone (Supplementary Fig. S2). A sensitivity analysis using highest rank across analytes for sample selection identified 3 clusters with a high overlap in participant cluster affiliation among original Clusters 1 and 2 with a lower agreement between methods observed in the smaller Cluster 3 (Supplementary Fig. S4).

**Table 1:** Baseline demographics across TDA clusters.

Characteristic	Total (N = 129)	Cluster 1 (N = 50)	Cluster 2 (N = 64)	Cluster 3 (N = 15)	p-value
**Male gender—no. (%)**	86(66.7%)	31(62%)	41(64.1%)	14(93.3%)	0.06^†^
**Age—years, median (IQR)**	41.3 (30.1, 56)	51.8 (37.3, 65)	37.1 (28.05, 49.55)	36.3 (24.6, 55.2)	< 0.001^ǂ^
**Race or ethnic group—no. (%)**					0.01^†^
White	81 (62.8)	30 (60.0)	43 (67.2)	8 (53.3)	
Black	31 (24.0)	16 (32.0)	12 (18.8)	3 (20.0)	
Other	6 (4.7)	1 (2.0)	5 (7.8)	2 (13.3)	
Asian	5 (3.9)	0 (0)	3 (4.7)	2 (13.3)	
Native American	3 (2.3)	0 (0)	1 (1.6)	0 (0)	
Native Hawaiian	3 (2.3)	3 (6.0)	0 (0)	0 (0)	
**Ethnicity—no. (%)**					0.88^†^
Hispanic or Latinx	31(24)	13(26)	15(23.4)	3(20)	
**Charlson Comorbidity Index (CCI)—median (IQR)**	0 (0, 2)	2 (2, 3)	0 (0, 0.5)	0 (0, 1)	0.009^ǂ^
**Body mass index—kg/m**^**2**^**, median (IQR)**	30 (27, 34)	33.5 (29, 37)	28 (25, 31)	28 (25, 31)	< 0.001ǂ
**Days post-symptom onset—median (IQR)**	21.0 (18.0, 25.0)	20 (17.0, 25.0)	21.0 (19.0, 25.5)	22.0 (21.0, 25.0)	0.16^ǂ^
**Hospitalization at timepoint—no. (%)**					< 0.001^ǁ^
ICU	10(7.8)	9(18)	1(1.6)	0	
Inpatient	28(21.7)	16(32)	7(10.9)	5(33.3)	
Outpatient	91(70.5)	25(50)	56(87.5)	10(66.7)	
**Peak severity—no. (%)**					< 0.001^ǁ^
Death	5 (3.9)	5 (10.0)	0 (0)	0 (0)	
ICU	16 (12.4)	10 (20.0)	3 (4.7)	3 (20.0)	
Inpatient	31 (24.0)	18 (36.0)	9 (14.1)	4 (26.7)	
Outpatient	77 (59.7)	17 (34.0)	52 (81.3)	8 (53.3)	
**Peak oxygen requirement—no. (%)**					< 0.001^ǁ^
None	87 (67.4)	23 (26.4)	55 (63.2)	9 (10.3)	
Low-flow nasal cannula	23 (17.8)	13 (48.1)	7 (10.9)	3 (20.0)	
HFNC or NIPPV	14 (10.9)	11 (40.7)	1 (1.6)	2 (13.3)	
Mechanical ventilation	5 (3.9)	3 (11.1)	1 (1.6)	1 (6.6)	
**Systemic steroid use—no. (%)**	5 (3.9)	5 (10.0)	0 (0)	0 (0)	

IQR: interquartile range; HFNC: high flow nasal cannula; NIPPV: non-invasive positive pressure ventilation.

^†^Chi-square test.

^ǂ^Mann Whitney U test.

^ǁ^Fischer’s Exact test.

**Figure 2:**
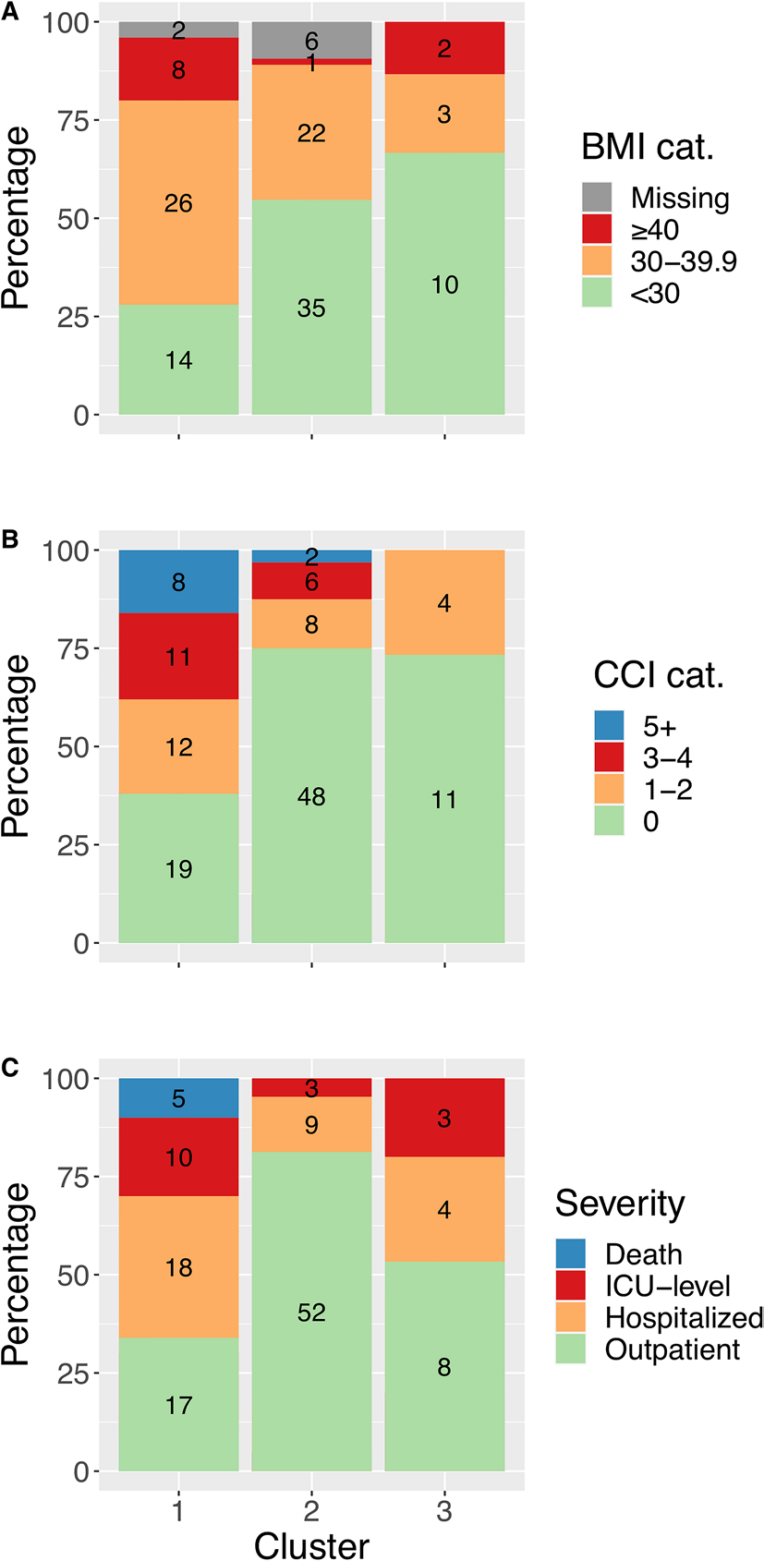
Cluster differences with bar plots (% [n]) of comorbid diseases and severity by cluster. (**A**) BMI (body mass index) category (range in kg/m^2^) prevalence by cluster; (**B**) Charlson Comorbidity Index (CCI) category prevalence by cluster; (**C**) peak levels of severity by cluster. Total (n) presented in the center of each category.

Age differed significantly between clusters (p < 0.001). Participants from Clusters 2 (median 37.1 years of age; IQR, 28.1 to 50.0) and 3 (median 36.3 years of age; IQR, 24.6 to 55.2) were younger than in Cluster 1 (median 51.8 years of age; IQR, 37.3 to 65.0) (Table 1). The prevalence of male gender was similar among Cluster 1 (62.0%, n = 31), Cluster 2 (64.1%, n = 41), and the general cohort (66.7%), but cluster 3 was predominantly male (93.3%, n = 14). The median time from symptom onset to sample collection was 21 days (IQR 18 to 25) and did not differ between clusters (Table 1). Peak disease severity, as categorized by hospitalization status, was also found to differ significantly among the clusters (p < 0.001). Cluster 1 had the highest prevalence of severe COVID-19, comprising 66.0% (n = 33) hospitalized participants, compared to 46.7% (n = 7) hospitalized participants in Cluster 3, and 18.8% (n = 12) hospitalized participants in Cluster 2 (Fig. 2, Table 1). All fatal cases (n = 5) were in Cluster 1. No individuals had received baricitinib or tocilizumab, and hydroxychloroquine use was limited to 2 individuals in Cluster 1. Receipt of systemic steroids at the time of blood collection was limited to 5 participants in Cluster 1 (10.0%; n = 5).

The median CCI differed (p = 0.009) among clusters ranging from 2 (IQR, 2 to 3) in Cluster 1 to 0 (IQR 0 to 0.5) in Cluster 2 and 0 (IQR, 0 to 1) in Cluster 3. Most participants in Cluster 2 (75.0%) and in Cluster 3 (73.3%) had a CCI of 0 compared to 38.0% of individuals in Cluster 1 (Fig. 2). Additionally, median BMI was higher in Cluster 1 (33.5 kg/m^2^; IQR, 29.0 to 37.0) compared to in Cluster 2 and Cluster 3, which were the same (28.0 kg/m^2^; IQR, 25.0 to 31.0) (Table 1). After adjusting for peak severity using logistic regression, participants with a higher BMI (OR: 1.1 per kg/m^2^, p = 0.002) and a higher CCI (OR: 1.3 for each score increase, p = 0.02) were more common in Cluster 1 compared to participants in Cluster 2 and 3 combined.

**Figure 3:**
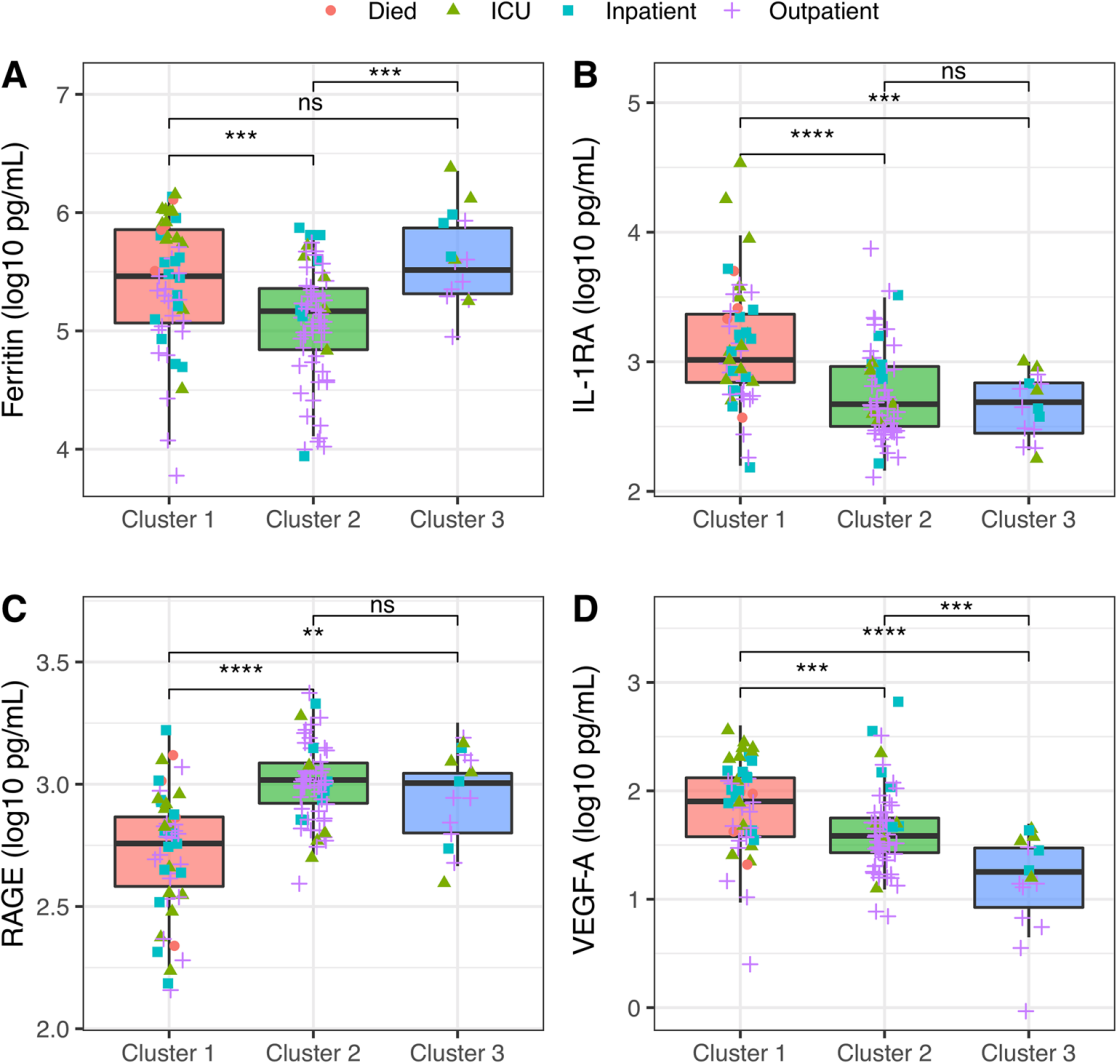
Box plots of markers selected in stepwise regression to identify characteristic biomarkers of each cluster: Ferritin (**A**), IL1RA (**B**), RAGE (**C**), and VEGFA (**D**) by cluster. Kruskal–Wallis test performed comparing analyte levels between clusters. **p ≤ 0.01; ***p ≤ 0.001; ****p ≤ 0.0001.

The distributions of each analyte were different across clusters using a chi-squared test, except for IL-5 and IFN-γ which had a similar distribution (Table 2). Certain biomarkers including CRP, IL-6, IL-1RA, d-dimer, TNFR1, and VEGF-A were more elevated in Cluster 1 compared to Clusters 2 and 3 (Table 2; Fig. 3; Supplementary Fig. S5). RAGE was lower in Cluster 1 compared to Clusters 2 or 3 and IFN-γ was lower in Cluster 1 compared to Cluster 2 (Fig. 3; Supplementary Fig. S5). Cluster 3, a young cluster with moderate severity, was found to have higher ferritin, procalcitonin, and CXCL10, and lower VEGF-A compared to Cluster 2, a similarly young cluster with mild illness.

**Table 2:** Comparison of the Ella biomarkers across TDA clusters.

Variable	Plasma log_10_ pg/mg, median (IQR)				p-value*
	Total	Cluster 1	Cluster 2	Cluster 3	
CRP	6.75 (6.09, 7.62)	7.33 (6.67, 7.98)	6.43 (5.89, 7.11)	6.29 (5.68, 7.13)	** < 0.001**
CXCL10	2.17 (1.95, 2.37)	2.28 (1.94, 2.53)	2.1 (1.88, 2.28)	2.19 (2.09, 2.51)	**0.02**
d-dimer	5.69 (5.34, 6.23)	6.06 (5.68, 6.85)	5.49 (5.27, 5.87)	5.68 (5.3, 6.52)	** < 0.001**
Ferritin	5.3 (4.98, 5.65)	5.46 (5.07, 5.87)	5.17 (4.82, 5.37)	5.51 (5.31, 5.9)	** < 0.001**
IFNγ	− 0.25 (− 0.45, 0)	− 0.3 (− 0.58, 0)	− 0.21 (− 0.39, 0)	− 0.27 (− 0.46, 0.02)	0.11
IL1Ra	2.85 (2.56, 3.11)	3.01 (2.84, 3.37)	2.67 (2.5, 2.97)	2.69 (2.4, 2.92)	** < 0.001**
IL5	− 0.56 (− 0.86, 0.34)	− 0.57 (− 0.95, − 0.4)	− 0.57 (− 0.81, − 0.31)	− 0.51 (− 0.85, − 0.24)	0.69
IL6	0.26 (0.01, 0.63)	0.52 (0.24, 1.1)	0.07 (− 0.13, 0.43)	0.26 (0.05, 0.6)	** < 0.001**
Procalcitonin	1.78 (1.63, 2)	1.92 (1.7, 2.25)	1.69 (1.59, 1.86)	1.92 (1.77, 2.1)	** < 0.001**
RAGE	2.93 (2.78, 3.04)	2.76 (2.58, 2.87)	3.02 (2.92, 3.09)	3 (2.8, 3.07)	** < 0.001**
TNFR1	3.03 (2.95, 3.18)	3.15 (3.01, 3.3)	2.98 (2.91, 3.08)	3.04 (2.97, 3.16)	** < 0.001**
VEGFA	1.64 (1.43, 1.92)	1.9 (1.57, 2.12)	1.58 (1.43, 1.76)	1.25 (0.8, 1.52)	** < 0.001**

For each subject, one sample was selected based on highest coefficient of variation.

*Distributions among all clusters compared using a Kruskal–Wallis test.

Significant values are in bold.

Stepwise regression, both unadjusted and adjusted for peak severity, was used to identify a subset of analytes that were most characteristic of each cluster (Supplementary Table S1). The distinguishing biomarker of Cluster 1 were relatively high IL-1RA and low RAGE levels; these subjects had a high severity phenotype compared to other clusters (Fig. 3; Supplementary Fig. S5; Supplementary Table S1). Regardless of peak severity, Cluster 2 was characterized by relatively low procalcitonin and high RAGE levels. Cluster 3 was characterized by low VEGF-A after peak severity adjustment (Fig. 3; Supplementary Fig. S5; Supplementary Table S1). When restricting the analysis to those not receiving steroids, the models were qualitatively unchanged, and the same covariates were selected. A sensitivity analysis adjusting for duration of symptoms also did not change the analytes selected and did not qualitatively change the results.

After adjustment for age, sex, and CCI, Cluster 1 was associated with a 5.22 (95% CI 1.31 to 20.80) increased odds of ICU-level illness or death (Supplementary Table S2). The AUROC for ICU-level illness or death increased with the addition of cluster designation from 0.78 to 0.83. However, the AUROC was similar when including clinical biomarkers (CRP, d-dimer, and ferritin) in the model (AUROC: 0.87) compared to clinical biomarker covariates with clusters included (AUROC: 0.88).

## Discussion

We demonstrated that a prospective cohort with a wide spectrum of disease severity can be stratified into three distinct inflammatory profiles using 12 plasma protein biomarkers during the middle phase of COVID-19. In contrast to most biomarker studies, our findings were drawn from a diverse multi-center cohort. Clusters stratified host phenotypes with different severity levels, demographics, and comorbid conditions. Combinations of biomarkers, independent of clinical information, grouped participants into one of three distinct clusters: high COVID-19 severity, older, with comorbid conditions (Cluster 1); low severity, younger, less comorbid illness (Cluster 2); and a moderate severity, younger, previously healthy, almost entirely male group (Cluster 3). A subset of biomarkers (i.e., IL1-RA, VEGF-A, and RAGE) were most representative of each cluster. Whilst exploratory, this reveals potential translational approaches to using host-biomarker stratification with advanced clustering and network analytical techniques to better understand what drives phenotypic differences in the clinical presentation of COVID-19.

Patterns of inflammation or cell injury observed for the different clusters could suggest dysregulated pathways associated with COVID-19 pathology. Cluster 1 was found to be the highest severity cluster with all fatal cases and most ICU-level cases. This cluster contained distinctly more subjects with baseline comorbid conditions and obesity as defined by BMI ≥ 30. While it is unsurprising that acute phase reactants were higher, Cluster 1 subjects had notably higher IL-1RA compared to Cluster 2 and 3, clusters represented by participants with less comorbid conditions. In contrast to Cluster 1, comorbid illness was uncommon, and the median age was over 15 years younger in Cluster 3. However, 7 of 15 participants were hospitalized in Cluster 3. While IL1RA levels were high in Cluster 1, IL1RA levels were lower in Cluster 3, which was similar to the mild Cluster 2. Consistent with this trend, prior work has identified IL-1RA as a potential mediator between obesity and COVID-19 severity^24^. IFN-γ was lower and IL-6 higher in Cluster 1 compared to the Cluster 2 participants. This pattern of an aberrant Th1 response has been previously identified to be associated with severe COVID-19 and potentially distinct from influenza infection^24^. Cluster 1 aligned with baseline comorbid illnesses known to be risk factors for severe COVID-19 with potentially distinct inflammatory cascade patterns demonstrated compared to Cluster 3.

Cluster 3 was unique in that it had a combination of low VEGF-A but had elevated ferritin and higher prevalence of severe illness compared to Cluster 2, a mild illness cluster with comparable demographics. While sample size is limited, 14 of 15 participants in Cluster 3 were male, suggestive of a biologic sex difference in immune response among these previously healthy young men. Sex differences leading to severe COVID-19 among men have been previously described with X-linked TLR7 deficiency^25,26^ and on a larger scale with sex-related differences in innate and T-cell responses^27^. A combination of low VEGF-A and elevated ferritin may identify a unique inflammation subtype and merits further study with external cohorts.

RAGE, a biomarker of acute lung injury^28^, was found to have different distributions between clusters. In contrast to prior research^29^, RAGE levels appeared to be higher among the younger and relatively milder COVID-19 severity Cluster 2 compared to Cluster 1. Compared to other clusters, RAGE was elevated along with IFN-γ in the less symptomatic Cluster 2, but with lower acute phase reactants ferritin and procalcitonin. The converse was true with Cluster 1 where lower levels of RAGE in individuals were noted, along with elevated acute phase reactants (i.e., CRP, procalcitonin, and ferritin). This association of lower RAGE with higher severity Clusters 1 and 3 contrasts with a direct association with COVID-19 mortality^30^. However, our results may differ by accounting for biomarker patterns rather than evaluating each biomarker in isolation and our study did not account for initial RAGE levels that may decrease over time^31^. It is possible that RAGE could be an adaptive anti-inflammatory protein in Cluster 2 during the middle phase. Soluble RAGE has been shown to reduce vascular injury in rodent models^32,33^ and could be protective against vascular inflammation mediated^34^. The paradoxically inverse relationship observed between RAGE and these commonly used acute phase reactants between the clusters merits further investigation into longitudinal changes.

While this study, to our knowledge, is the first to use a network clustering approach to understand relationships between biomarker patterns and heterogenous clinical phenotypes of COVID-19, there are limitations worth noting. Samples were collected from April 2020 to January 2021 and treatment practices and epidemiologic changes over time may have affected inflammation patterns. Hence, we incorporated a sensitivity analysis excluding those that received systemic steroids in Cluster 1 to aid in interpreting the findings. In addition, while results were drawn from a diverse multi-center cohort, the sample size may limit our ability to identify uncommon biomarker patterns and external validation is needed of patterns identified. Additionally, regression was used to adjust for peak severity to identify biomarker and comorbid condition associations with clusters distinct from severity trajectory differences. While this is a novel feature of this biomarker study, residual confounding related to peak severity remains possible. Lastly, the phases of illness likely vary between individuals. While this cross-sectional look at the middle phase was able to identify major differences in inflammation patterns, additional approaches with larger sample sizes could identify shifts in phenotypes over time or to identify trajectory phenotypes using techniques such as latent class analysis^35,36^. For example, all participants that received corticosteroids were in Cluster 1. This could be due to persistent breakthrough inflammation, but future research with dynamic models is needed to explore this further. Despite limitations, results presented here are hypothesis generating and should be evaluated further in additional cohorts.

This approach constitutes an early exploratory step in identifying host biomarker patterns that may be leveraged for personalized interventions, and offers new insights for COVID19 prognosis, therapy, and prevention with techniques that could be extended to understanding other severe infections. Using analytes identified from our international sepsis cohort research^6^, 3 biomarker clusters with different phenotypic associations were identified among those with heterogenous COVID-19 presentations. Inclusion of inflammation biomarkers including IL-1RA, VEGF-A, and RAGE should be considered for future mediation analyses to identify precision biomarkers to guide COVID-19 therapeutics. The application of these biomarkers derived from non-COVID-19 severe infection research suggests that pathogen-agnostic sepsis biomarkers could be identified for personalized approaches to triage of care or immunomodulation strategies. Further validation of these markers and clustering algorithms with external cohorts could inform point-of-care biomarker assay development to guide more individualized approaches to COVID-19 care.

## Supplementary Information

Supplementary Legends.

Supplementary Figure S1.

Supplementary Figure S2.

Supplementary Figure S3.

Supplementary Figure S4.

Supplementary Figure S5.

Supplementary Table S1.

Supplementary Table S2.

Supplementary Information.

## Data Availability

Completely de-identified data may be provided upon reasonable request to the corresponding author. Identifying data that would compromise privacy could not be shared publicly.
