# Supplementary material for: Distinct blood inflammatory biomarker clusters stratify host phenotypes during the middle phase of COVID-19
Source: Sci Rep. 2022 Dec 28;12:22471. doi: 10.1038/s41598-022-26965-7 (PMC9795438; doi:10.1038/s41598-022-26965-7)
Supplement: Supplementary file 1 — Supplementary Legends. [file 41598_2022_26965_MOESM1_ESM.docx]

**Supplementary Figure Legend**

**Supplementary Figure S1.** Scatter plot with plasma IL6 (A), ferritin (B), and CRP (C) over time with LOESS (locally estimated scatterplot smoothing) curves stratified by peak severity from EPICC cohort between 0 to 29 days post symptom onset. While participants with mild illness have a down sloping LOESS curve, participants with moderate or severe illness had a late peaking curve during the third and fourth week of illness (the inflammatory phase). Each point represents a sample level with some participants having multiple samples collected. Points are jittered to avoid overplotting.

**Supplementary Figure S2.** A: Spearman’s correlation matrix of inflammation biomarkers with size and color scale for correlation coefficient; B: PCA showing dimensionality of different analytes; C: PCA showing dimensionality of different analytes with peak severity labeled by color

**Supplementary Figure S3.** TDA networks were generated for a range of resolution settings to examine the persistence of subject clusters and their interrelatedness. Clusters 1 (blue), 2 (red), and 3 (green) were observed consistently throughout different resolution and gain settings in the EurekaAI Workbench platform (SymphonyAI, Los Altos, CA, USA). Each node represents a combination of 12 plasma protein analyte levels and its size increases with the number of participants that are included.

**Supplementary Figure S4.** TDA networks were compared to determine agreement between TDA clusters based on coefficient of variation (CoV) and a sensitivity analysis with ranked sample selection. The percent agreement between methods was determine and was high for Clusters 1 and 2.

**Supplementary Figure S5.** Box plots of inflammatory biomarkers divided by TDA cluster. Each sample with peak COVID severity labeled by color and shape. Kruskal-Wallis test performed comparing analyte levels between clusters. *: p ≤ 0.05; **: p ≤ 0.01; ***: p ≤ 0.001; ****: p ≤ 0.0001
