# Supplementary figures and images for: Distinct blood inflammatory biomarker clusters stratify host phenotypes during the middle phase of COVID-19
Source: Sci Rep. 2022 Dec 28;12:22471. doi: 10.1038/s41598-022-26965-7 (PMC9795438; doi:10.1038/s41598-022-26965-7)

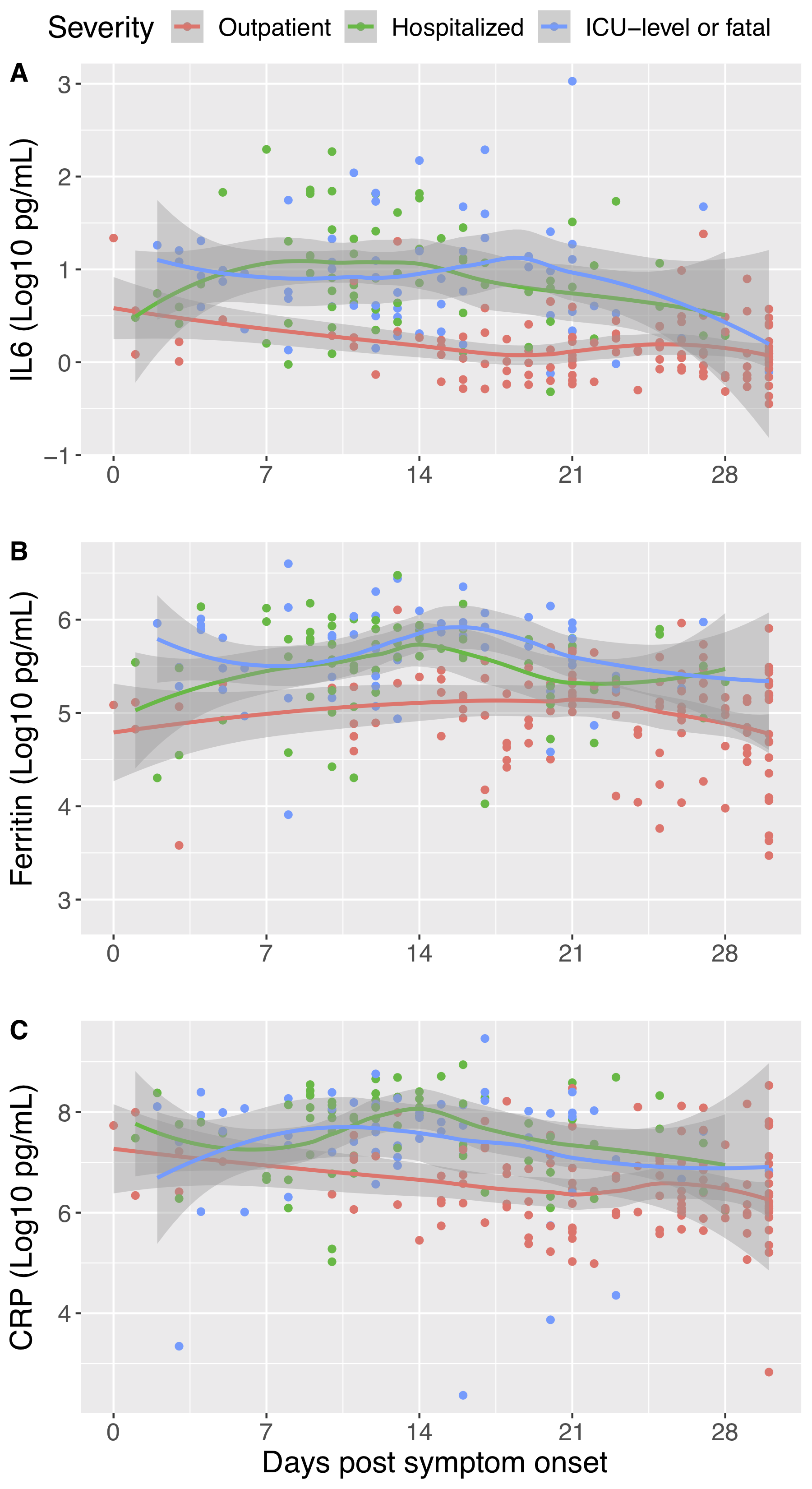

Supplement: Supplementary file 2 — Supplementary Figure S1. [file 41598_2022_26965_MOESM2_ESM.tiff]

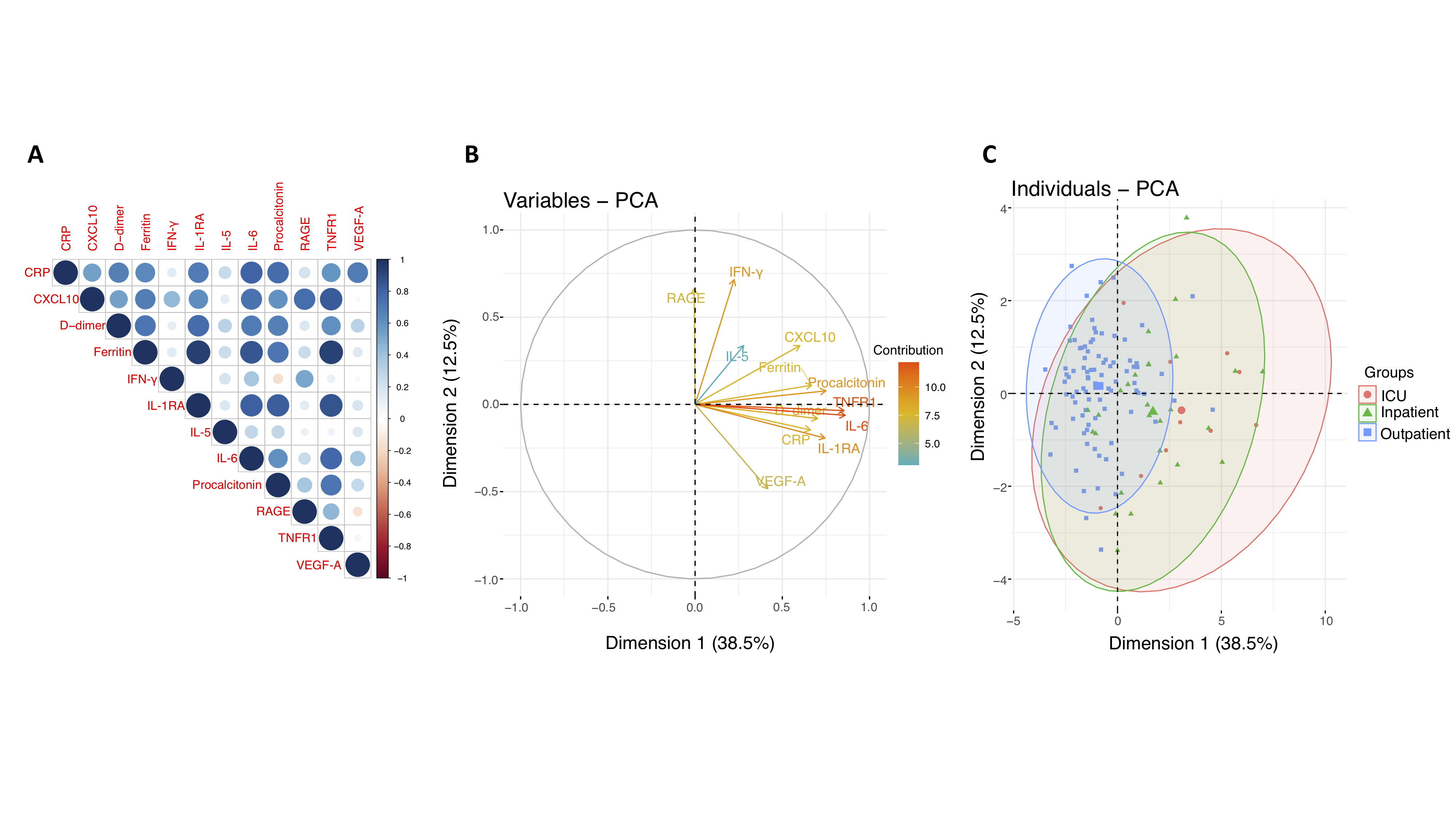

Supplement: Supplementary file 3 — Supplementary Figure S2. [file 41598_2022_26965_MOESM3_ESM.tiff]

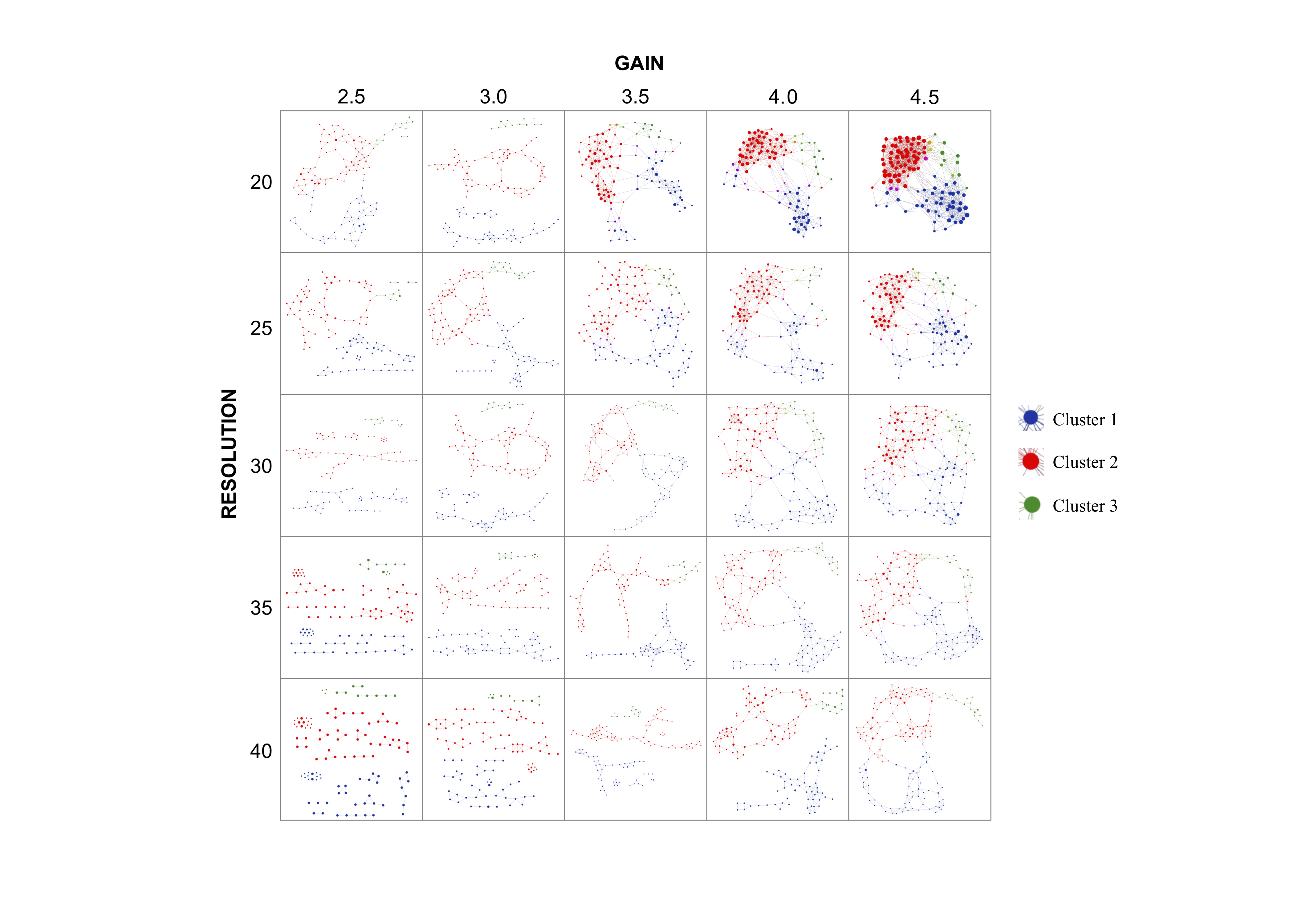

Supplement: Supplementary file 4 — Supplementary Figure S3. [file 41598_2022_26965_MOESM4_ESM.tiff]

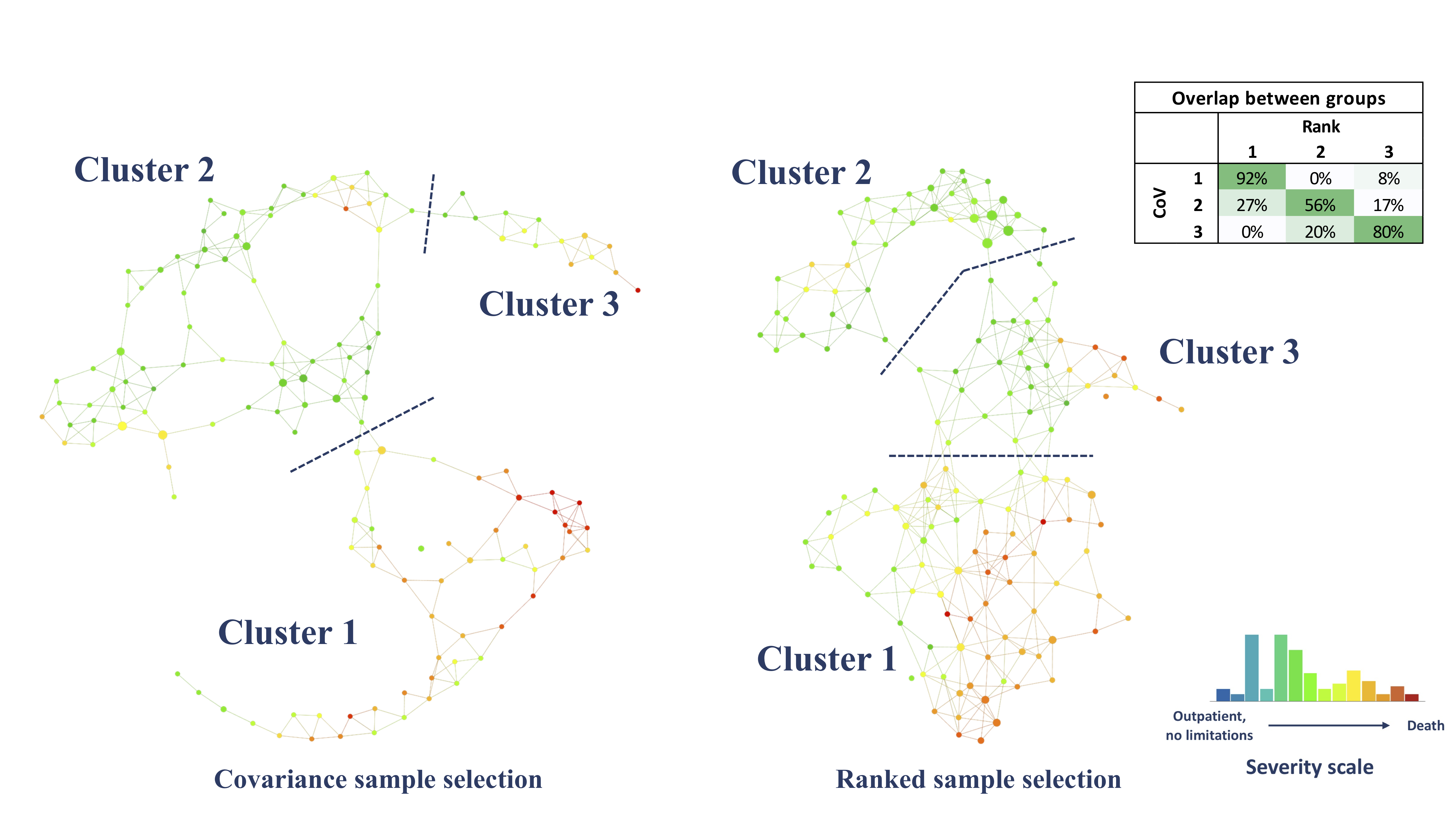

Supplement: Supplementary file 5 — Supplementary Figure S4. [file 41598_2022_26965_MOESM5_ESM.tiff]

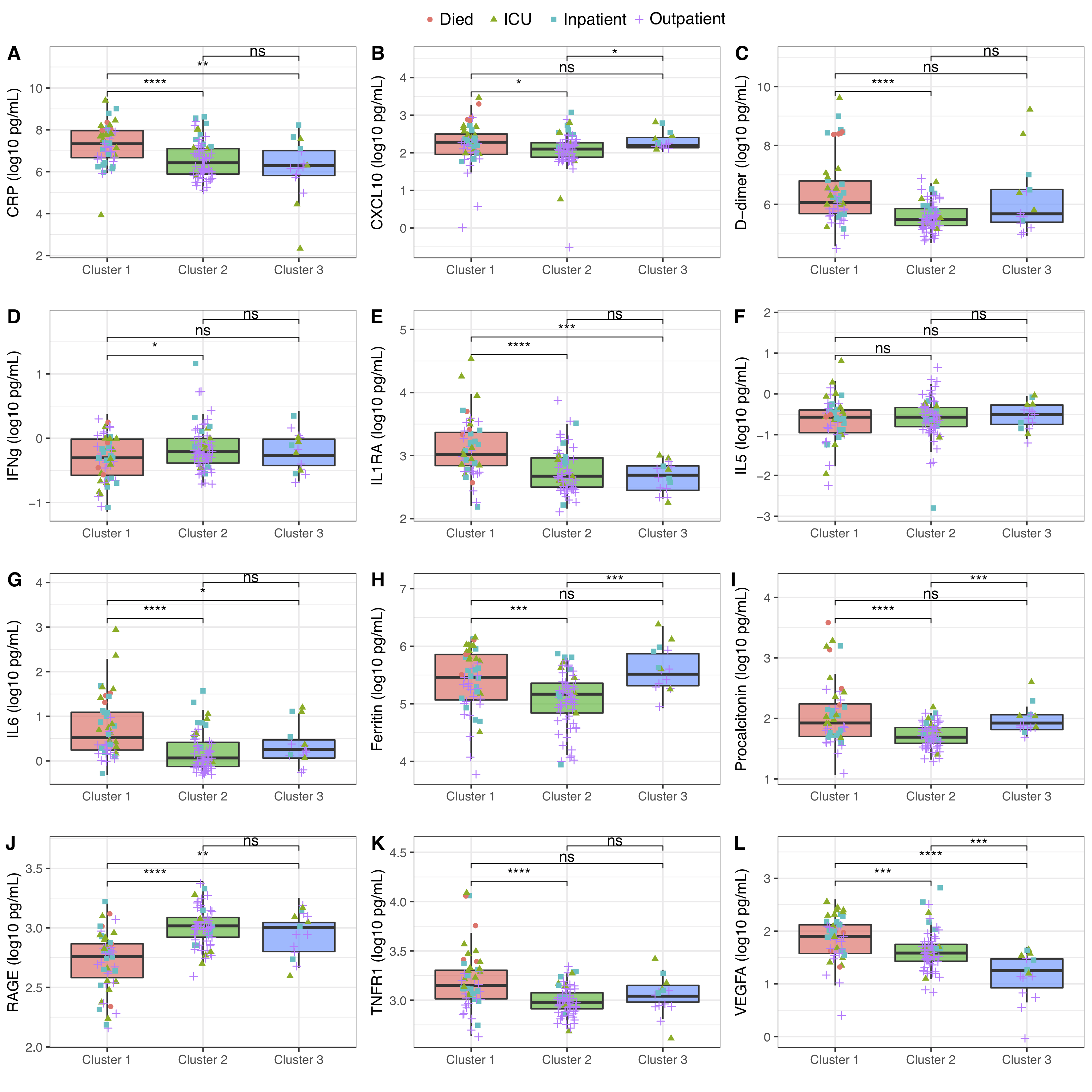

Supplement: Supplementary file 6 — Supplementary Figure S5. [file 41598_2022_26965_MOESM6_ESM.tiff]
