# Supplementary material for: Distinct blood inflammatory biomarker clusters stratify host phenotypes during the middle phase of COVID-19
Source: Sci Rep. 2022 Dec 28;12:22471. doi: 10.1038/s41598-022-26965-7 (PMC9795438; doi:10.1038/s41598-022-26965-7)
Supplement: Supplementary file 7 — Supplementary Table S1. [file 41598_2022_26965_MOESM7_ESM.docx]

**Table S1.** Multivariable logistic regression models for clusters and plasma immune biomarker levels. Models were fit comparing each cluster against all other clusters.

| Cluster | | Covariates in unadjusted model | OR* (95% CI) | AIC | Covariates in severity-adjusted** model | OR* (95% CI) | AIC |
| --- | --- | --- | --- | --- | --- | --- | --- |
| 1 |  | |  | 87.7 |  |  | 85.4 |
|  | CXCL10 | | 238.1 (6.1, 9,238.2) |  | Severity | 4.9 (2.0, 11.9) |  |
|  | IL1RA | | 4.3 (2.1, 8.9) |  | IL1RA | 4.1 (2.1, 8.2) |  |
|  | RAGE | | 0.0007 (0.00005, 0.01) |  | RAGE | 0.001 (0.00008 0.02) |  |
| 2 |  | |  | 91.9 |  |  | 102.0 |
|  | Ferritin | | 1.0 (0.990, 0.997) |  | Severity | 0.4 (0.1, 0.9) |  |
|  | Procalcitonin | | 2.5e-14(1.7e-22, 3.6e-06) |  | Procalcitonin | 2.8e-14 (06.5e-22, 1.2e-06) |  |
|  | RAGE | | 839.1 (58.7, 11,991.9) |  | RAGE | 217.0 (24.2, 1,948.7) |  |
| 3 |  | |  | 58.7 |  |  | 73.4 |
|  | Ferritin | | 1.0 (1.002, 1.007) |  | Severity | 1.9 (0.9, 3.9) |  |
|  | VEGFA | | 3.1e-46 (3.2e-72, 2.9e-20) |  | VEGFA | 1.1e-33 (2.6e-53, 4.8e-14) |  |
| AIC: Akaike information criterion  *Estimates are in ng/ml scale. | | | | |  |  |  |
| **Peak severity on an ordinal scale with outpatient-level= 0, inpatient-level=1, ICU-level care =2, death =3 | | | | | | | |
