# Supplementary material for: Distinct blood inflammatory biomarker clusters stratify host phenotypes during the middle phase of COVID-19
Source: Sci Rep. 2022 Dec 28;12:22471. doi: 10.1038/s41598-022-26965-7 (PMC9795438; doi:10.1038/s41598-022-26965-7)
Supplement: Supplementary file 8 — Supplementary Table S2. [file 41598_2022_26965_MOESM8_ESM.docx]

**Table S2.** Multivariable logistic regression models for ICU admission and death.

| Model | Covariates | | OR* (95% CI) | AIC | AUROC |
| --- | --- | --- | --- | --- | --- |
| 1 | Age (years) | | 1.05 (1.00, 1.11) | 104.09 | 0.78 |
|  | Female sex | | 0.53 (0.167, 1.70) |  |  |
|  | Charlson comorbidity index | | 1.11 (0.74, 1.66) |  |  |
| 2 | Age (years) | | 1.04 (0.99, 1.10) | 100.84 | 0.83 |
|  | Female sex | | 0.55 (0.16, 1.86) |  |  |
|  | Charlson comorbidity index | | 1.12 (0.75, 1.69) |  |  |
|  | TDA Cluster 1 | | 5.22 (1.31, 20.80) |  |  |
|  | TDA Cluster 2 | | REF |  |  |
|  | TDA Cluster 3 | | 5.22 (0.85, 32.15) |  |  |
| 2 | Age (years) | | 1.04 (0.98, 1.10) | 91.45 | 0.87 |
|  | | Female sex | 0.81 (0.21, 3.20) |  |  |
|  | | Charlson comorbidity index | 1.12 (0.73, 1.72) |  |  |
|  | | CRP | 0.74 (0.35, 1.58) |  |  |
|  | | D-dimer | 1.56 (0.87, 2.85) |  |  |
|  | | Ferritin | 13.76 (1.46, 129.73) |  |  |
| 3 | | Age (years) | 1.03 (0.97, 1.10) | 93.78 | 0.88 |
|  | | Female sex | 0.81 (0.20, 3.26) |  |  |
|  | | Charlson comorbidity index | 1.09 (0.71, 1.69) |  |  |
|  | | CRP | 0.69 (0.31, 1.52) |  |  |
|  | | D-dimer | 1.44 (0.78, 2.65) |  |  |
|  | | Ferritin | 13.46 (1.33, 136.3) |  |  |
|  | | TDA Cluster 1 | 2.61 (0.55, 12.33) |  |  |
|  | | TDA Cluster 2 | REF |  |  |
|  | | TDA Cluster 3 | 1.30 (0.16, 10.93) |  |  |
| AIC: Akaike information criterion; AUROC: Area under receiver operating characteristic curve  *Biomarker estimates are in ng/ml scale. | | | | | |
