# Supplementary material for: Distinct blood inflammatory biomarker clusters stratify host phenotypes during the middle phase of COVID-19
Source: Sci Rep. 2022 Dec 28;12:22471. doi: 10.1038/s41598-022-26965-7 (PMC9795438; doi:10.1038/s41598-022-26965-7)
Supplement: Supplementary file 9 — Supplementary Information. [file 41598_2022_26965_MOESM9_ESM.docx]

*TDA networks*

All TDA networks were generated using a normalized Pearson correlation similarity matrix and Symphony AyasdiAI’s “Neighborhood” lens function. This function generates an embedding of high-dimensional data into two dimensions with a k-nearest neighbor graph of the data. Examining TDA structures at different resolutions and gains helps determine which structures are robust or may be artifact(7). The range of available resolution and gain (range from 1 to 10, in 0.1 intervals) settings are dictated by the size of the data set and the graphing algorithm. Resolution can range from 1 node to 1 node per sample. We examined the dataset under a range of TDA resolution and gain settings, and 3 clusters were identified with stable and consistent features.

*Ella protein analytes*

Analytes were on the same custom cartridges when possible, depending on dynamic range: 1. IL-5, RAGE, and VEGF-A together at 1:2 dilution; 2. IFN-γ, IL-6, procalcitonin, and TNFR1 together at 1:2 dilution; 3. D-dimer, ferritin, and IL-1RA at 1:10 dilution. CXCL10 was run alone at a dilution of 1:2 dilution. CRP was run alone at 1:2000 and re-run with dilutions up to 20,000 as needed dilution.
